# Protein Engineering Strategies to Expand CRISPR-Cas9 Applications

**DOI:** 10.1155/2018/1652567

**Published:** 2018-08-02

**Authors:** Lucas F. Ribeiro, Liliane F. C. Ribeiro, Matheus Q. Barreto, Richard J. Ward

**Affiliations:** ^1^Department of Biology, Faculdade de Filosofia, Ciências e Letras de Ribeirão Preto, University of São Paulo, São Paulo, SP, Brazil; ^2^Department of Biochemistry and Immunology, Faculdade de Medicina de Ribeirão Preto, University of São Paulo, São Paulo, SP, Brazil; ^3^Department of Chemistry, Faculdade de Filosofia, Ciências e Letras de Ribeirão Preto, University of São Paulo, São Paulo, SP, Brazil

## Abstract

The development of precise and modulated methods for customized manipulation of DNA is an important objective for the study and engineering of biological processes and is essential for the optimization of gene therapy, metabolic flux, and synthetic gene networks. The clustered regularly interspaced short palindromic repeat- (CRISPR-) associated protein 9 is an RNA-guided site-specific DNA-binding complex that can be reprogrammed to specifically interact with a desired DNA sequence target. CRISPR-Cas9 has been used in a wide variety of applications ranging from basic science to the clinic, such as gene therapy, gene regulation, modifying epigenomes, and imaging chromosomes. Although Cas9 has been successfully used as a precise tool in all these applications, some limitations have also been reported, for instance (i) a strict dependence on a protospacer-adjacent motif (PAM) sequence, (ii) aberrant off-target activity, (iii) the large size of Cas9 is problematic for CRISPR delivery, and (iv) lack of modulation of protein binding and endonuclease activity, which is crucial for precise spatiotemporal control of gene expression or genome editing. These obstacles hinder the use of CRISPR for disease treatment and in wider biotechnological applications. Protein-engineering approaches offer solutions to overcome the limitations of Cas9 and generate robust and efficient tools for customized DNA manipulation. Here, recent protein-engineering approaches for expanding the versatility of the *Streptococcus pyogenes* Cas9 (SpCas9) is reviewed, with an emphasis on studies that improve or develop novel protein functions through domain fusion or splitting, rational design, and directed evolution.

## 1. Introduction

Progress in genetic engineering, metabolic engineering, and synthetic biology will be determined by the development of versatile, user-friendly technologies for the precise and efficient manipulation of cells. The clustered regularly interspaced short palindromic repeat- (CRISPR-) associated protein 9 (CRISPR-Cas9) system is a particularly attractive tool for gene/base editing, gene regulation studies, epigenetic modulation, genome imaging, and manipulation of chromatin topology [1–7]. This technology has attracted considerable attention due to its simplicity for targeting and modifying a specific DNA sequence. Use of the Cas9 protein circumvents the requirement of other DNA-binding proteins such as zinc finger nucleases (ZFNs) and transcription activator-like effector (TALE) to be reengineered each time in order to bind different target DNA sequences. In CRISPR-Cas9, the element that specifies the DNA target is not the protein itself, but a single-guide RNA (sgRNA) molecule, which is straightforward to design and to synthesize [8]. The sgRNA-Cas9 complex binds to the DNA to create a double-stranded break. Cleavage by the sgRNA-Cas9 requires both sequence complementarity between the sgRNA (spacer sequence) and the target DNA sequence (“protospacer”) as well as the presence of an appropriate protospacer-adjacent motif (PAM) sequence at the 3′ end of the protospacer sequence [9]. CRISPR-Cas9 is part of the adaptive immune system in prokaryotes, and the PAM sequence allows this immune system to distinguish between self and nonself DNA target [10]. In addition, there is significant variation in PAM specificity between Cas9 orthologs [11], which extends the applications of Cas9 to complex systems that require orthogonality or multiplexing [12].

**Figure 1:**
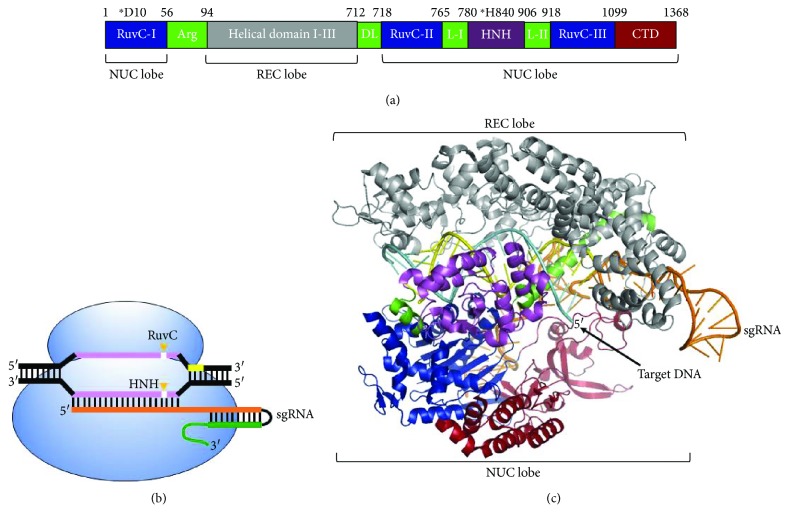
Overall organization, structure, and function of CRISPR-associated protein 9 (Cas9) from *Streptococcus pyogenes* (SpCas9). (a) Schematic representation of the domain organization of the SpCas9. Asterisks denote catalytical residues. (b) Cas9 (blue) requires a sgRNA that has a 20 bp region complementary to the target DNA. Cas9 requires two RNA components—CRISPR RNA (crRNA; orange) and transactivating RNA (tracrRNA; green). sgRNA is a chimeric RNA in which crRNA and tracrRNA are fused through a linker. PAM sequence (5′-NGG-3′) is shown in yellow and is crucial for binding and cleavage. DNA cleavage occurs in two different domains: the HNH domain that cuts the target strand and RuvC domain that cleaves the nontarget strand. (c) Cartoon representation of the crystal structure of SpCas9 (PDB 4UN3). Cas9 domains are colored according to the scheme in (a). Abbreviations: Arg: arginine-rich bridge helix; DL: disordered linker; CTD: C-terminal domain; NUC: nuclease lobe; PAM: protospacer-adjacent motif; REC: recognition lobe.

The Cas9 enzyme is an endonuclease found in some bacteria and Archaea [13–15]. The Cas9 of *Streptococcus pyogenes* (SpCas9) is the best characterized and most widely applied for DNA sequence manipulation. The SpCas9 is comprised of 1368 amino acids and is organized in multiple domains each with a distinct function (Figure 1(a)) [16]. The HNH-like and RuvC-like domains have nuclease activity and are so named because they present sequence similarity to other endonucleases. The HNH-like domain cleaves the DNA strand that is complementary to the sgRNA (target strand), and the RuvC-like domain cleaves the noncomplementary DNA strand (nontarget strand) (Figure 1(b)) [8, 17, 18]. Alanine substitution of key residues in the RuvC domain (D10A) produces a nick in the targeting strand, while the H840A mutation of the HNH domain nicks only the nontargeting strand. The double D10A/H840A mutant eliminates nuclease activity in both domains producing a catalytically inactive, or “dead,” Cas9 (dCas9) [8]. Nevertheless, dCas9 retains the ability of Cas9 to bind specifically to a target DNA sequence.

The structure of Cas9 presents two lobes, an *α*-helical recognition lobe (REC) and a nuclease lobe (NUC) (Figures 1(a) and 1(b)) [16]. The REC is comprised of three *α*-helical domains and has no structural similarity to any other known protein. The NUC lobe includes the nuclease domains RuvCs, HNH, and also the C-terminal domain (CTD). The two lobes are connected by two linker sequences, the arginine-rich bridge helix (Arg) and the disordered linker (DL). The sgRNA-DNA complex is bound at the interface between the two lobes. The CTD domain contains the PAM-interaction residues necessary for the recognition of the PAM sequence, in which arginine residues at positions 1333 and 1335 play an important role [19]. The apo Cas9 protein (without sgRNA) adopts an inactive conformation, in which the PAM-interaction region is largely disordered [20], and sgRNA binding is a key event for Cas9 activation [20]. When bound to sgRNA, the REC lobe of Cas9 undergoes a large conformational change and the Cas9-sgRNA complex is ready to probe for the target DNA sequence. Single-molecule experiments have demonstrated that the recognition of target DNA by Cas9-sgRNA occurs through three-dimensional collision [21]. The process starts by probing the correct PAM sequence; if PAM is not found, the protein rapidly dissociates from the DNA. When PAM is present, the DNA adjacent to the PAM sequence begins to denature and subsequent base pairing of the sgRNA forms a RNA:DNA hybrid [21, 22]. DNA cleavage requires perfect complementarity between the sgRNA and 10–12 nucleotides located at the 3′ end of the 20-nt spacer sequence, and this hybrid structure is denominated as the “seed” region [8, 21, 23, 24]. Imperfect base pairing outside the seed region can be tolerated and results in off-target Cas9 activity [25]. This detailed understanding of the structural basis of Cas9 activity is the starting point for improving and expanding the functions and applications of this protein.

Despite its proven potential, CRISPR-Cas9 system has limitations that restrict its wide use for disease treatment or in other biotechnological applications. These limitations include the strict dependence of a PAM sequence, off-target DNA cleavage, and problems for CRISPR delivery posed by the large size of the protein [26–30]. Not only could protein engineering offer elegant approaches to overcome these limitations but may also be used to improve the kinetic and biophysical properties of the enzyme. Engineered Cas9 could provide robust and efficient tools for DNA manipulation tailored to specified applications. In this review, we will discuss recent protein-engineering approaches to expand Cas9 functions, emphasizing those studies that have improved or created SpCas9 functions through domain fusion or splitting, rational design, and directed evolution.

## 2. Engineering Cas9 by End-to-End Fusion or Protein Domain Insertion

**Figure 2:**
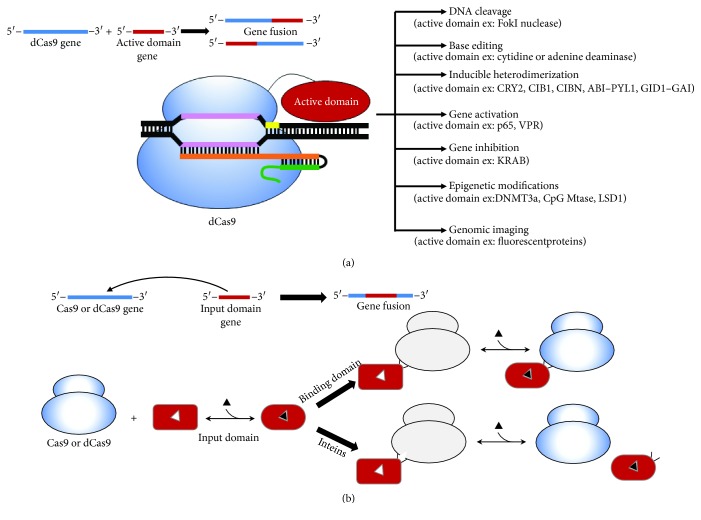
Schematic representations of Cas9 engineering by domain fusion. (a) End-to-end gene fusion approach. A diverse range of new technologies for DNA manipulation can be created by end-to-end fusion of Cas9 with an active domain. These fusions have been used to expand Cas9 applications. (b) Schematic depiction of Cas9 engineering by domain insertion. A switch behaviour could emerge in such a way that the Cas9 protein could be regulated by the input domain's recognition of an input signal. Either binding domains or inteins have been used to generate Cas9 with a switch response. DNA sequences are depicted as lines. A light gray color of the Cas9 indicates that the protein is inactive or less active while blue represents the active state. The signal that modulates the switch is showed as a black triangle.

Protein domains are evolutionarily conserved polypeptide units that generally show independent structural or functional properties. Proteins containing multiple domains represent more than two-thirds of the proteins found in prokaryotes and eukaryotes [31]. Moreover, protein domains can act as structural reservoirs to generate new protein architectures [32]. Bioengineers have used domains as building blocks to generate new proteins with novel biotechnological applications [33–38]. Multidomain proteins can be generated by either end-to-end fusion or by domain insertion. End-to-end fusion consists of creating a peptidyl linkage between the N-terminal residue of one domain and the C-terminal residue of another. Since no prior knowledge of the protein structure is necessary, end-to-end fusion is amongst the most widely used strategies in protein engineering [37], and several studies have used this approach to engineer Cas9 with the aim of manipulating its properties for different applications (Figure 2(a)) [39–42].

Alternatively, multidomain proteins can be formed by domain insertion in which one domain (insert) is spliced into another domain (acceptor) either at a specific position or by random insertion. Domain insertion can create structural coupling among the combined domains, with the emergence of new functions (Figure 2(b)) [43]. Furthermore, in contrast to end-to-end fusion in which proteins are linked by a single contact point, when two domains are fused by domain insertion, they will be linked by peptidyl bonds at two contact points, which can generate more stable structures [36, 44–46]. In the following examples, we discuss current strategies for engineering SpCas9 through end-to-end fusion and domain insertion [43].

### 2.1. Reducing Off-Target Events

One of the main limitations of the CRISPR-Cas9 system is the high level of off-target DNA-cleavage events [47]. In order to improve specificity, dimerization-dependent Cas9-based nucleases have been created by end-to-end fusion of dCas9 with a dimerization-dependent FokI nuclease domain (Figure 2(a)) [48, 49]. The fusion protein doubled the sequence length required for target DNA recognition and efficient cleavage, resulting in a >140-fold higher specificity in human cells as compared to wild-type dCas9 [49]. However, this strategy restricts the overall targetable sequence space because it requires two Cas9-compatible sequences to be close enough (~15 to 25 bp) in order for *Fok*I to dimerize and cleave the target DNA. In an alternative strategy to improve Cas9 DNA-binding precision [41], a DNA-binding domain (either ZFP (zinc finger proteins) or a TALE (transcription activator-like effector)) was fused to a Cas9 in which key PAM-interacting residues were mutated to reduce DNA-binding affinity. Three different Cas9-ZFP end-to-end fusion proteins were designed to bind to 12 base pair sequences, and their activities were tested at previously defined off-target sites. The approach not only dramatically decreased off-target cleavage events but the Cas9-ZFP chimeras also reduced the size of the engineered protein, which is advantageous for the viral delivery systems.

In a further attempt to decrease the off-target events, the SpCas9 was fused to the structurally unstable protein domain: dihydrofolate reductase (DHFR) or the estrogen receptor (ER50) [42], which target the fusion protein for rapid proteasomal degradation [50]. Switched systems were created by fusion of DHFR or ER50 to both the N- and C-termini of SpCas9. These fusion proteins bind to the DNA target with full endonuclease activity only in the presence of trimethoprim (TMP) or 4-hydroxytamoxifen (4-HT), small molecules that stabilize DHFR and ER50, respectively. In the absence of TMP or 4-HT, fused proteins were targeted for degradation by the proteasome [42]. This approach demonstrates that limiting Cas9 action to a short and controlled period may decrease off-target activity.

### 2.2. Base Editing with Cas9 Fusion Chimeras

Cas9 has also been fused end-to-end with cytidine deaminase [51] and adenine deaminase [52] (Figure 2(a)) in order to develop nucleotide base editors. In both cases, the goal was to efficiently correct point mutations related to diseases, without the generation of random insertions and deletions (indels). To achieve this goal, dCas9 was used as a DNA-binding domain and fused to a deaminase. In 2016, Komor et al. created a so-called “base editor” to convert cytidines into uridine within a sequence of five nucleotides located between the protospacer and PAM [51]. A cytidine deaminase from Rat known as APOBEC1 (apolipoprotein B mRNA editing enzyme, catalytic polypeptide-like 1) was fused to the N-terminus of dCas9 using a linker in order to maintain deaminase activity. Iterative optimizations in the linker and chimera were performed, and each step of optimization produced a new “generation” of the construction. Therefore, the third generation of base editors (BE3) consisted of APOBEC1 linked to the N-terminus of dCas9 with the catalytic His840 restored and a uracil glycosylase inhibitor linked at Cas9 C-terminus (APOBEC–linker–dCas9(A840H)–UGI). This chimera showed a permanent point mutation correction of up to 75% of total cellular DNA with 1% indel formation [51]. Since the use of this chimera is limited by off-target activity, the BE3 has been improved by incorporating four point mutations (N497A, R661A, Q695A, and Q926A) to generate a high-fidelity base editor (HF-BE3) with reduced off-target activity [53]. However, efficient CRISPR-Cas9-based editors require a 5′-NGG-3′ PAM, limiting the sequence space in the genome that can be efficiently targeted. Furthermore, these base editors are only able to efficiently convert C to T within ~5 bp of the editing window, and this window could introduce undesired changes around the base target. In an attempt to address these limitations, SpCas9 was substituted for dCas9 from *Staphylococcus aureus* (SaCas9) in the BE3 to generate the APOBEC1–SaCas9n–UGI (SaBE3). In order to obtain a narrower editing window, a series of iterative optimizations were conducted resulting in base editors with altered PAM specificities (YE1-BE3, EE-BE3, YE2-BE3, or YEE-BE3) [54]. Although mutations in the cytidine deaminase enzyme could narrow the editing window, the variants were not able to discriminate among the cytidines within the window. An alternative base-editing chimera has been described that uses an engineered human APOBEC3a (eA3A) domain in the BE3 [55]. The eA3A-BE3 chimera was able to correct a mutation in a human beta-thalassemia promoter with >40-fold higher precision than BE3. Furthermore, the eA3A-BE3 had lower off-target activity as compared to BE3 [55].

Using an alternative approach, Gaudelli et al. sought to develop a base editor to convert A:T base pairs to G:C by fusing dCas9 to an adenosine deaminase [52]. However, since there is no enzyme known to deaminate adenine in DNA, the first step was to evolve *E. coli* TadA, a tRNA adenine deaminase that converts adenine to inosine. Mutations in the vicinity of the D108 residue in TadA were sufficient to introduce adenine deamination activity against DNA substrates. The TadA A106V/D108N mutant was fused to the N-terminus of the Cas9 (D10A mutant with nickase activity) [52]. After several rounds of iterative optimization, evolved fused proteins were capable of converting A:T base pairs into G:C base pairs in human cells with approximately 50% efficiency, high specificity (~99%), and low rates of indel formation (<0.1%). These studies highlight both the potential and the importance of improving the precision of base editors using CRISPR-Cas9 for use in medical therapies.

### 2.3. Cas9 Chimeras for Controlling Transcriptional Regulation Activated by Light

Several studies have reported the use of CRISPR-Cas9 to control gene expression [40, 56–58]. Two similar systems have been developed by fusion of CRISPR-Cas9 and a light-inducible heterodimerizing cryptochrome 2 (CRY2) together with the calcium- and integrin-binding protein 1 (CIB1) (Figure 2(a)) [40, 56]. Nihongaki et al. fused dCas9 with CIB1 and CRY2 with a transcriptional activator [56]. In this system, dCas9 is expressed and binds to a target DNA sequence guided by the gRNA. Upon blue light irradiation, CRY2 and CIB1 heterodimerize, and the transcriptional activator is recruited to the target locus, activating gene expression. After optimization, a fusion protein was generated in which the N-terminus of dCas9 was linked to a nuclear localization signal (NLS), and the C-terminus of dCas9 was linked to CIB1 with a truncated C-terminus. The activator includes three NLS for correct nuclear localization, the photolyase homology region of CRY2 (CRY2PHR), and the activator domain (p65)-NLSdCas9-trCIB1 and (NLS)_3_-CRY2PHR-p65, and the final fusion protein demonstrated a 31-fold induction. Polstein and Gersbach created a light-activated CRISPR/Cas9 effector (LACE) system in which a different fusion protein combination was used [40]. Optimized LACE consisted of two CIB domains fused to both the N- and C-termini of Cas9 together with a CRY2 fused to a transcriptional activator. This system was used in HEK293T cells to control expression of human IL1RN and presented a 400-fold increase after 30 hours of blue light irradiation.

**Figure 3:**
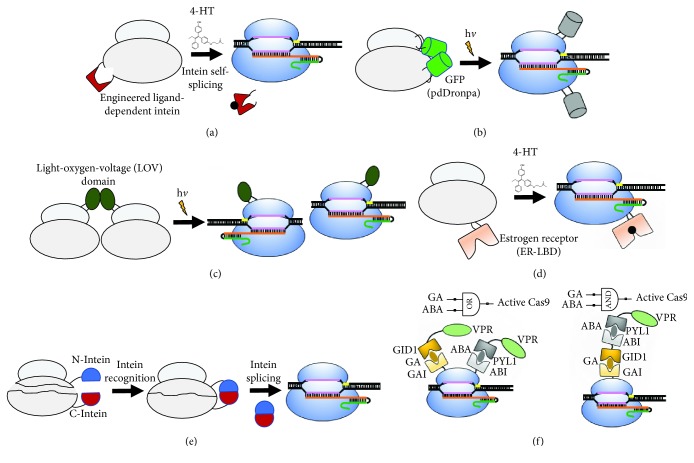
Selected case studies of engineering dCas9 by domain fusion. (a) Intein insertion inactivates dCas9, and in the presence of 4-HT, the inserted intein (red) undergoes self-splicing and restores the active Cas9 structure and function [70]. (b) Insertion of an engineered GFP (pdDronpa) that dimerizes in the dark and prevents DNA binding. On light illumination, pdDronpa dissociates and enables the binding of dCas9 to DNA [71]. (c) In the absence of light, the LOV domain (dark green) maintains a stable dimer that sterically blocks dCas9 function. Light induces LOV dissociation and Cas9 activation [74]. (d) Domain insertion of a ligand-binding domain (LBD) of the estrogen receptor ER leads to an allosteric activation by 4-HT [75]. Rather than domain insertion, an end-to-end fusion with ER-LBD results to nuclear translocation by 4-HT [58]. (e) A split Cas9 composed of two separate fragments is fused to intein sequences that perform self-splicing upon dimerization, leading to fully active Cas9 [83]. (f) The dCas9 fusion with multiple domains can generate a multi-input system and produce logic gates [57]. A VPR–SpdCas9 construct induces gene expression in the presence of gibberellin (GA) OR/AND abscisic acid (ABA). A light gray color of the Cas9 indicates that the protein is inactive or less active while blue represents the active state.

In addition to light activation, systems for chemical activation of dCas9 to regulate gene expression have been developed [57, 58]. A variant of Cas9 with nuclease activity modulated by the presence of the tamoxifen analogue 4-HT (Figure 3(d)) [58] was based on fusions of human estrogen receptor 2 (ERT2) domain at either the N- or C-terminus of Cas9, in which the positions of the nuclear localization signal (NLS) was varied. The result was a switch protein working as a rapidly inducible conditional genome-editing system, with high DNA-cleavage efficiency after induction with 4-HT and low background in the absence of inducer [58]. Variants of Cas9 responding to both chemical and light inducers have also been developed [57] where an activator domain (VPR) was fused to six chemical and light-inducible heterodimerization domains (Figure 2(a)): abscisic acid- (ABA-) inducible ABI–PYL1, gibberellin- (GA-) inducible GID1–GAI, rapamycin-inducible FKBP–FRB, phytochrome-based red-light-inducible PHYB–PIF, cryptochrome-based blue-light-inducible CRY2PHR–CIBN, and light-oxygen-voltage-based blue-light-inducible FKF1–GI [57]. The best results were obtained with ABA- and GA-dimerized VPR–SpdCas9-activated systems (Figure 3(f)), in which EGFP protein expression increased 165- and 94-fold, respectively, upon induction. These efficiencies were similar to those obtained for the VPR-dCas9 direct fusion protein, demonstrating the potential for the use of these chimeras as gene expression regulators. The platform developed in this work was also created to be used as AND, OR, NAND, and NOR dCas9 logic gates, offering a protein-based alternative to produce a functional output from multiple inputs [57].

### 2.4. Epigenetic Regulation by Cas9 Chimeras

Cas9 chimeras have also been created with the aim of controlling and manipulating epigenetic modifications (Figure 2(a)) [59–62]. Fusion of a DNA-binding domain to a cytosine DNA methyltransferase has been a common strategy in order to elucidate the effects of DNA methylation in mammalian cells, in which dCas9 was fused to a DNA methyltransferase domain named DNMT3a [59–63]. In these chimeras, the dCas9 acts as a DNA-binding domain and directs binding to a specific DNA promotor target determined by the sgRNA control. The methyltransferase then modulates DNA methylation of the target promoter resulting in the downregulation of target genes.

In a different strategy, DNMT3a was artificially split generating N- and C-terminal fragments. Subsequently, the DNMT3a C-terminal fragment was fused to the C-terminus of dCas9 using a 15-amino-acid linker [20]. The dCas9 directed the assembly of methyltransferase fragments at the CpG site, resulting in an efficient (~70%) and predictable DNA methylation [63]. Application of Cas9 fusions for epigenetic manipulation also includes the fusion of a histone demethylase LSD1 (Lys-specific histone demethylase) to Cas9 from *Neisseria meningitides* [64]. This chimera was used to target the distal enhancer region of the endogenous transcription factor gene Oct4 in mouse embryonic stem cells, resulting in the repression of Oct4 expression and loss of pluripotency [64]. In a separate study, targeting three endogenous promoters using a chimera in which the C-terminus of a dCas9 was fused to the highly conserved acetyltransferase p300 resulted in acetylation of the histone H3 lysine 27 at the target site and transcriptional activation of target genes [65]. Finally, dCas9 was fused to two families of proteins directly involved in gene silencing through methylation, the KRAB (Krüppel-associated box) that forms a complex with two histone methyltransferases, and the DNMT [59]. This study showed that the combination of dCas9-KRAB with dCas9-DNMT improved silencing efficiency. These examples highlight the versatility of the Cas9 as a tool for silencing transcription and targeting regulatory regions.

### 2.5. Cas9 Chimera for Genome Imaging

With the aim of simplifying the study of spatial genome organization, fusion of fluorescent protein domains to dCas9 has been applied to the visualization of genomic loci and chromatin spatiotemporal dynamics in live cells (Figure 2(a)) [39, 66, 67]. Although the fusion of single-color protein fluorescent labels (eGFP) to SpdCas9 has been described [39, 66], multiple labels are required to differentiate interchromosomal and intrachromosomal loci within the nucleus. The design of multicolor versions of dCas9 from three bacterial orthologs, *S. pyogenes*, *N. meningitides*, and *Streptococcus thermophiles*, has been reported [67]. Each dCas9 ortholog, targeting the human telomere DNA repeat, was fused to a fluorescent protein (green fluorescent protein (GFP), red fluorescent protein (RFP), or blue fluorescent protein (BFP)). The construction NLS-dCas9-(NLS)_1–3_-(XFP)_3_, in which XFP means GFP, RFP, or BFP, permitted the estimation of the intranuclear distance between loci in different chromosomes and the linear distance between two loci in the same chromosome, allowing assessment of the DNA compaction in these regions in a live cell. The development of these tools may enable the study of the 4D nucleome and the regulation of gene expression in different eukaryotic cell types and at various stages of development and differentiation [67].

### 2.6. Cas9 Chimeras Created by Domain Insertion

A limitation of many Cas9-derived systems is that its activity is not directly modulated, and domain insertion has been used to create new functions in Cas9, including inducible response. Fine tuning of the Cas9 is of key importance to reduce the off-target activity and to allow a spatial-temporal control of the protein. Inducible Cas9 can be created by fusing two domains in such a way that the Cas9 activity is regulated by the recognition of an input signal by a sensor domain.

Inteins (intervening proteins) are proteins that perform protein-splicing posttranslational modifications [68] with the goal of creating a regulated Cas9; SpCas9 has been recombined with the intein 37R3-2 [69, 70]. Since inteins are self-excised from the “immature” polypeptide producing a functional mature protein, the insertion of an intein into the Cas9 may lead to its inactivation, and its excision may result in Cas9 activation. The 37R3-2 intein was engineered to perform protein splicing only in the presence of chemical 4-HT. Subsequently, the engineered 37R3-2 was inserted at 15 positions of Cas9 distributed throughout its different domains. Insertions at Ala728, Thr995, and Ser1154 did not lead to a significant inactivation of Cas9 in the absence of 4-HT, suggesting that these positions tolerate large protein insertions. Insertions at positions S219 and C574 produced low nuclease activity in the absence of 4-HT and an ~4-fold increase of this activity in the presence of the compound (Figure 3(a)). The on-target/off-target ratio was 6-folds higher on average and up to 25-folds higher when compared to the wild-type Cas9, demonstrating that the insertion of the intein into specific sites of Cas9 was able to increase the protein precision for gene editing. However, a decrease in nonhomologous end joining (NHEJ) efficiency was observed, and in addition, the activation of the Cas9 activity by the intein domain is irreversible.

### 2.7. Photoregulation of Cas9 Chimeras Created by Domain Insertion

The ideal tool for DNA editing would be an inducible Cas9 that could be readily modulated by the presence of a nontoxic, nonmetabolizable, inexpensive signal. A recent study explored the photomodulated dimerizing protein domains known as pdDronpa [71], a green fluorescent protein which dimerizes in the dark but dissociates to monomers upon illumination with light at 500 nm [72]. A single-chain photoswitchable Cas9 (ps-SpCas9) was engineered by insertion of the pdDronpa protein into Cas9 at two positions, and in the absence of light, the dimerization of the pdDronpa protein blocks the binding of Cas9 to the DNA (Figure 3(b)). The fluorescent protein was inserted into two loops in Cas9 at the REC2 domain and into the CTD domain of the NUC lobe. These loops are situated across the DNA-binding cleft and are occluded by pdDronpa dimerization. Although psCas9 produced an indel level lower than the parental Cas9 that was similar to other photoinduced two-component Cas9 systems [73], the ps-SpCas9 functions as a single chain, and the pdDronpa domain can also be used as a localization and expression marker. The ps-SpCas9 was also engineered to regulate gene transcription, for which the mutations D10A and H841A were introduced in Cas9 to create ps-dSpCas9, which was then fused to a VP64-p65-Rta (VPR) transactivation module [57] at the N-terminus. The pdDronpa domain was replaced to a new version of the protein called pdDronpa1.2 with reduced basal activity in the absence of light [72]. This new protein, denominated as VPR-ps-dSpCas9, showed a 58-fold induction of the reporter gene (mCherry) after light illumination. This is higher than an optimized multiple-chain light-activated Cas9 effector (LACE) system [40, 56] and comparable with chemically inducible systems [57].

A recent study also used a photomodulated dimerizing protein known as RsLOV, a photoreceptor from *Rhodobacter sphaeroides*, which in the dark is a homodimer and upon illumination by blue light dissociates to its monomeric form [74]. The RsLOV was inserted using flexible linkers at 231 positions throughout the dCas9 protein via multiplex-inverse PCR (Figure 3(c)). Chimeras were selected by fluorescence-activated cell sorting (FACS) based on the ability to modulate RFP expression in the presence of blue light (470 nm), and two photoactivatable RsLOV-Cas9 variants demonstrated repression activity enhancement under blue light. A temperature-sensitive variant, denoted as tsRC9, also was isolated and presented activity at 29°C but negligible activity at 37°C [74].

### 2.8. Exploring the Limits of Insertional Fusion with Cas9

In an elegant approach, Oakes et al. [75] have characterized SpCas9 tolerance to domain insertion by random insertion of an engineered Mu transposon flanked by BsaI endonuclease restriction sites into dCas9. Deep sequencing analysis showed that the transposon was inserted in >70% of all possible amino acid sites of dCas9, and this library was used as a starting point for insertion of the 86 aa PDZ domain. The library containing the plasmid carrying dCas9 and Mu transposon was digested with BsaI, and the transposon was replaced by the BsaI-digested gene encoding the PDZ domain with flanking amino acid linkers. After PDZ insertion, FACS was used to identify dCas9 variants that could repress RFP expression, and 127 positions were identified as tolerant to PDZ insertion. These sites tended to cluster around flexible loops, at solvent-exposed residues and the ends of helices, and preferential sites for domain insertion were found mainly within the helical II recognition lobe (REC), in the RuvC-III region and throughout the CTD domain. Insertions into vital motifs, such as sgRNA-binding grooves, the bridge helix, the PAM-binding pocket, and the DNA/RNA heteroduplex annealing channel, resulted in impaired Cas9 functionality. Subsequently, eight insertion sites were chosen to introduce a SH3 domain, of which six showed repression levels similar to parental dCas9 [75]. Furthermore, 2–4 insertions of multiple PDZ and SH3 domains were introduced into dCas9 at validated insertion sites, and many of these constructs were capable of repressing expression to a degree comparable with the parental dCas9. Moreover, with the aim of developing a switch Cas9, the well-characterized human estrogen receptor-*α* ligand-binding domain (ER-LBD) was inserted into the naive dCas9 transposition library (Figure 3(d)). The ER-LBD binds 4-HT, and a dCas9 variant carrying an ER-LBD insertion (denominated as darC9:231) capable of modulating gene repression in the presence of 4-HT was identified. The catalytic residues (D10 and H840) were reintroduced in darC9 to produce arcC9:231. In the presence of 4-HT, arC9 increased chromosomal cleavage 100- and 24-fold in *E. coli* and human cells, respectively [75]. These results demonstrate that the domain insertion profiling of Cas9 can generate important functional information, which can be used to facilitate protein engineering.

## 3. Structure-Based Cas9 Engineering

The crystal structure of Cas9 from *S. pyogenes* [76] provides the basis for rational engineering and modification of the protein. Using structure-based design principles, it is possible to enhance specific properties of Cas9, reduce nonspecific activity, switch specific regions of the protein, and obtain enzymes that are suitable for specific genome-engineering applications.

Nonspecific cleavage of DNA is the consequence of imperfect complementarity between the RNA guide and a genomic site, leading to off-target gene editing. The stabilization of the nontarget DNA by Cas9 is through the positively charged groove located between the HNH-, RuVC-, and PAM-interacting residues in the CTD domain. Engineering of this region holds the promise of reducing off-target edition, and the substitution of the 32 positively charged residues by alanine in this groove leads to the identification of five residues that decreased off-target cleavage [77]. The combination of these mutations generated three Cas9 variants with normal on-target activity and decreased off-target indel formation. Using a similar approach, alanine substitution of four polar or charged residues located in the same groove resulted in undetectable levels of off-target indels [78]. Structural analysis revealed that these residues participate in nonspecific interactions with the phosphate backbone of the target DNA strand.

The cleavage of DNA by Cas9 is dependent of the recognition of the protospacer-adjacent motif (PAM). The recognition of a specific PAM sequence by Cas9 is one of the limitations of using the wild-type SpCas9. Sequence databases presently contain over 1000 Cas9 orthologs whose different PAM specificities could provide insights to engineer the SpCas9 and alter its specificity [79]. The CTD domain (containing the PAM-interacting motif) from the SpCas9 has been exchanged for the Cas9 orthologous CRISPR-3 from *Streptococcus thermophilus* [76], which altered the recognition of the 5′-NGG-3′ to the 5′-NGGNG-3′ PAM from *S. thermophilus*.

A limitation of SpCas9 in gene therapy is its size (~4.3 kb), which limits its efficient gene-based delivery via recombinant adenoassociated virus (rAAV) [80, 81]. rAAV is a viral vector that has been successfully used in therapeutic gene editing. However, the size limit of an insert in this vector is ∼4.7 kb, which is problematic for packaging SpCas9, sgRNA, and control elements [80, 81]. To reduce the size of Cas9, the effects of deletion of specific Cas9 domains on protein activity were investigated [76]. It was observed that the enzyme with the REC2 domain deleted (Δ175–307) maintained 50% activity as compared to its wild-type counterpart, indicating that this domain is not critical for DNA cleavage and could potentially be targeted to reduce the size of Cas9 [76].

Using another approach, the nuclease (M1 to E57-GSS linker-G729 to D1368) and the *α*-helical lobes (G56 to S714) of Cas9 were expressed separately [82], and although neither of the two fragments presented activity, both parts readily reassemble to an active form on contact with the sgRNA, albeit with reduced editing efficiency. In another study, the Cas9 coding sequence was divided into two segments, and each was fused to an intein moiety (N-moiety: M1 to E573 and C-moiety: C574 to D1368 or N-moiety: M1–K637 and C-moiety: T638–D1368) [83]. After the sequences were introduced into the cell by a recombinant adenoassociated virus, protein splicing efficiently reconstituted the Cas9 as a single polypeptide with full activity (Figure 3(e)), suggesting a means to use CRISPR for gene therapy via adenovirus release of genetic material. Splitting Cas9 into two segments (N-moiety: D2–V713 and C-moiety: S714–D1.368) and fusing each segment to a photodimerization domain (pMag and nMag) [84] resulted in reassociation of the two fragments on irradiation with blue light and restoration of Cas9 structure and activity, suggesting the possibility for optical regulation of Cas9 activity. Based on crystal structure analysis, the Cas9 was split into eleven sites [85], and in each case, the fragments were fused to FKBP or FRB that dimerize in the presence of rapamycin. In addition, the N-terminal fragment was fused to a nuclear export sequence (NES) and C-terminal fragment to a nuclear localization sequence (NLS). Thus, split Cas9 can be reassembled upon rapamycin induction and acted in the nucleus without detectable off-target activity. Moreover, a split dCas9 version was fused to the VP64 transactivation domain, and this construction was able to activate transcription of target genes in the presence of rapamycin.

The crystal structure of the Cas9 shows that the K866 undergoes substantial conformational change upon sgRNA binding, allowing the proper positioning of the target DNA. With the aim of modulation of Cas9, a site-specific photocaged lysine was introduced at these positions to create optochemical control of Cas9 [71]. The photocaged lysine at this position deactivated Cas9, and the caging group could be removed through light exposure. The photocaged Cas9 showed minimal background activity in the absence of UV light and reached parental Cas9 levels after light irradiation.

## 4. Engineering PAM Specificity by Directed Evolution

The sequences recognized by Cas9 are limited by the requirement of a specific protospacer-adjacent motif (PAM) [24]. One strategy to improve CRISPR-Cas9 is by changing PAM specificity using directed evolution techniques [86]. Based on the crystal structure of Cas9 [76], the PAM-interacting domain of *S*pCas9 was subjected to random mutagenesis, and a library of mutated sequences was screened against the 5′-NGA-3′ PAM-target site. Three Cas9 variants called VQR (D1135V/R1335Q/T1337R), EQR (D1135E/R1335Q/T1337R), and VRER (D1135V/G1218R/R1335E/T1337R) were obtained, whose PAM specificity changed from 5′-NGG-3′ observed in the wild-type Cas9 to 5′-NGA-3′, 5′-NGAG-3′, and 5′-NGCG-3′, respectively, thereby broadening the target range of the Cas9 [86].

This approach is limited because it needs to evolve each variant separately with a potential PAM sequence, and this limitation is exacerbated for Cas9 orthologs that specify longer PAMS. As an alternative, variants with relaxed specificities within the PAM could be evolved [87], and the predicted PI-domain from SaCas9 was randomly mutagenized and tested for PAM specificity, resulting in a Cas9 variant called KKH (E782K/N968K/R1015H) which showed the same DNA-cleavage specificity when compared to its wild-type counterpart (5′-NNGRRT-3′). Nevertheless, the KKH enzyme can also identify nonspecific sites such as 5′-NNARRT-3′, 5′-NNCRRT-3′, and 5′-NNTRRT-3′, which increases its targeting range. The crystal structures of the three variants [76] revealed that PAM-reprogramming of these enzymes is based on a synergistic effect of the mutations in the displacement of the PAM duplex, enabling different nucleotide sequences to be recognized by these new PAMs.

In order to enhance acquisition of spacer sequences flanked by non-NGG PAM motifs, SpCas9 variants were created by error-prone PCR, and after selection, a Cas9 variant I473F was identified with higher specificity for the PAM sequence 5′-NAG-3′ [88]. This variant caused an enhanced immune response against viruses due to higher rates of spacer acquisition, showing the importance of the enzyme not only in DNA-cleavage events but also in gaining new spacer sequences to facilitate the CRISPR immune response in bacteria.

A recent strategy that has been employed to broaden PAM specificity was to use phage-assisted continuous evolution (PACE) to evolve a SpCas9 variant (xCas9) [89]. Using PACE, hundreds of generations of directed evolution could be implemented, and screening used a bacterial one-hybrid selection in which dCas9 was fused to the *ω* subunit of a bacterial RNA polymerase which on binding to a PAM causes phage propagation. It was proposed that the replication of variants with broader PAM specificity would be favored. After continuous *in vivo* protein evolution, several variants were enriched in the pool, including R324L, S409I, and M694I, which are located near the DNA-sgRNA interface in the crystal structure. Restoring the catalytic residues showed that the variants were able to cleave the DNA with five PAM sequences (5′-NG-3′, 5′-NNG-3′, 5′-GAA-3′, 5′-GAT-3′, and 5′-CAA-3′), and off-target tests showed a significant off-target activity reduction as compared to the parental SpCas9.

## 5. Future Perspectives

Cas9 has a tremendous utility for the regulation and modification of complex biological systems. However, overcoming the limitations of the system is paramount to realize its full potential. An ideal Cas9-based tool should bind and/or cleave a single specific target in a complex genome without generating off-targets as side products. In addition, it would be highly desirable to develop Cas9 technologies endowed with precise spatiotemporal control, rapid responses to inducers, lack of toxicity, ease of customization, and high efficiency of *in vitro* and *in vivo* delivery. The studies described here showed that over recent years, new ways to improve Cas9 functions have been developed. These protein-engineering efforts have made significant contributions to the improvement of gene therapy, genomic imaging, and the emerging field of synthetic biology.

However, new Cas9-based tools are required in order to overcome a number of features that are still poorly explored. For example, most of the systems described in this review still present low delivery efficiency and off-target activity, are relatively expensive, and are not readily adapted for multiplexing. This suggests that there is still plenty of scope for engineering Cas9. For example, some inducible Cas9 systems use expensive steroids that have short half-lives in solution and relatively high toxicity for both prokaryote and eukaryote cells [90–92]. Engineering Cas9 to respond to a range of inexpensive and nontoxic signals would result in CRISPR applications that are more flexible and economically feasible on a larger scale. In addition, many of these studies are specific to mammalian cells. The adaptability of these tools to other platforms such as bacteria, fungus, and plant cells would greatly increase its biotechnological impact. Moreover, little is known about the allergenic potential of Cas9 in gene therapies in humans, and protein engineering plays a key role for studying and solving this potential issue. Protein engineering of catalytic and biophysical properties is as yet little explored, and Cas9 with improved catalytic efficiency or able to work in extreme conditions could be useful for engineering extremophilic organisms. In addition to Cas9 engineering, sgRNA engineering also has been used to enhance CRISPR functionality, and the combination of both approaches could be used to expand Cas9 applications [4, 93]. Given the rapid development of diverse engineered SpCas9s, it is likely that in the future, a wide range of tailored Cas9, working as simple or multiplex tools, will be available for a wide variety of genome-editing applications, including gene therapy and treatment of disease.

## Abbreviations

SpCas9: Cas9 from *Streptococcus pyogenes*
SaCas9: Cas9 from *Staphylococcus aureus*
PAM: Protospacer-adjacent motif
NLS: Nuclear localization signal
CRY2: Light-inducible heterodimerizing cryptochrome 2
CIB1: Calcium- and integrin-binding protein 1
4-HT: 4-Hydroxytamoxifen
VPR: Chimeric activation domain composed of the activation domainsVP64, P65, and Rta
pdDronpa: Engineered GFP that dimerizes in the dark
LOV: Photomodulated dimerizing protein
ER-LBD: Ligand-binding domain of the estrogen receptor.
